# Ki67 index in intrinsic breast cancer subtypes and its association with prognostic parameters

**DOI:** 10.1186/s13104-019-4653-x

**Published:** 2019-09-23

**Authors:** Atif Ali Hashmi, Kashif Ali Hashmi, Muhammad Irfan, Saadia Mehmood Khan, Muhammad Muzzammil Edhi, Javaria Parwez Ali, Shumaila Kanwal Hashmi, Huda Asif, Naveen Faridi, Amir Khan

**Affiliations:** 1 Liaquat National Hospital and Medical College, Karachi, Pakistan; 2 Nishtar Medical College Hospital, Multan, Pakistan; 3 Brown University, Providence, RI USA; 4 CMH Institute of Medical Sciences, Multan, Pakistan; 5 Kandahar University, Kandahar, 3802 Afghanistan

**Keywords:** Breast cancer, Intrinsic subtypes of breast cancer, Ki67 index, ER, PR, Her2neu

## Abstract

**Objectives:**

Ki67 is the most commonly used marker to evaluate proliferative index in breast cancer, however no cutoff values have been clearly defined for high ki67 index. Cancer management should be according to loco-regional profile; therefore, we aimed to determine ki67 index in 1951 cases of intrinsic breast cancer subtypes and its association with other prognostic parameters in our set up.

**Results:**

Triple negative breast cancers showed highest ki67 index (mean 50.9 ± 23.7%) followed by Her2neu (mean 42.6 ± 21.6%) and luminal B cancers (mean 34.9 ± 20.05%). Metaplastic and medullary breast cancers significantly showed higher ki67 index as compared to ductal carcinoma, NOS. No significant association of ki67 index was noted with any of the histologic parameters in different subtypes of breast cancer expect for tumor grade. Although, ki67 index is a valuable biomarker in breast cancer, however no independent prognostic significance of ki67 could be established in our study.

## Introduction

Transition from phenotypic to intrinsic molecular breast cancer subtypes has made a paradigm shift in breast cancer treatment. With advent of new treatment regimens, it becomes important to individualize therapy according to biomarker status of the tumor. Hormone receptor and human epidermal growth factor receptor 2 (her2neu) statuses impart both prognostic and predictive impact on breast cancer management. Therefore performing estrogen receptor (ER), progesterone receptor (PR) and her2neu biomarker studies has become standard of care in breast cancer management as per American Society of Clinical Oncology (ASCO) guidelines [1]. Markers of elevated proliferation generally indicate a poor outcome in any cancer. Over the past years, there is a considerable debate over the performance and interpretation of proliferative index markers like thymidine labeling index, S-phase fraction determined by flow cytometry and immunohistochemistry (IHC). Overall, proliferative index determined by IHC correlates well with S phase fraction measured by flow cytometry [2]. Although, there is still no consensus over an optimal cutoff value used to decide chemotherapy, but several studies found that high ki67 index is associated with higher rate of relapse and worse breast cancer survival [3, 4]. It is widely accepted that cancer management should be according to loco-regional profile and therapeutic protocols should be devised accordingly, however no large-scale cancer registry is available in this part of the world. Moreover ki67 may serve as a useful marker in tailoring treatment regimen as response to chemotherapy may be altered by the proliferative activity of cancer cells [5]. Therefore we aimed to determine the ki67 in newly defined intrinsic breast cancer subtypes and its association with other prognostic parameters in our set up.

## Main text

### Materials and methods

The study included 1951 cases of primary breast cancers. All of these patients underwent treatment at Liaquat National hospital during January 2011 till December 2016. An approval from institutional ethical review committee was taken before conducting the study. The specimens were of trucut biopsies, breast conservative surgical specimens (wide local excision) with sentinel lymph node dissection and modified radical mastectomies (MRM).

**Fig. 1:**
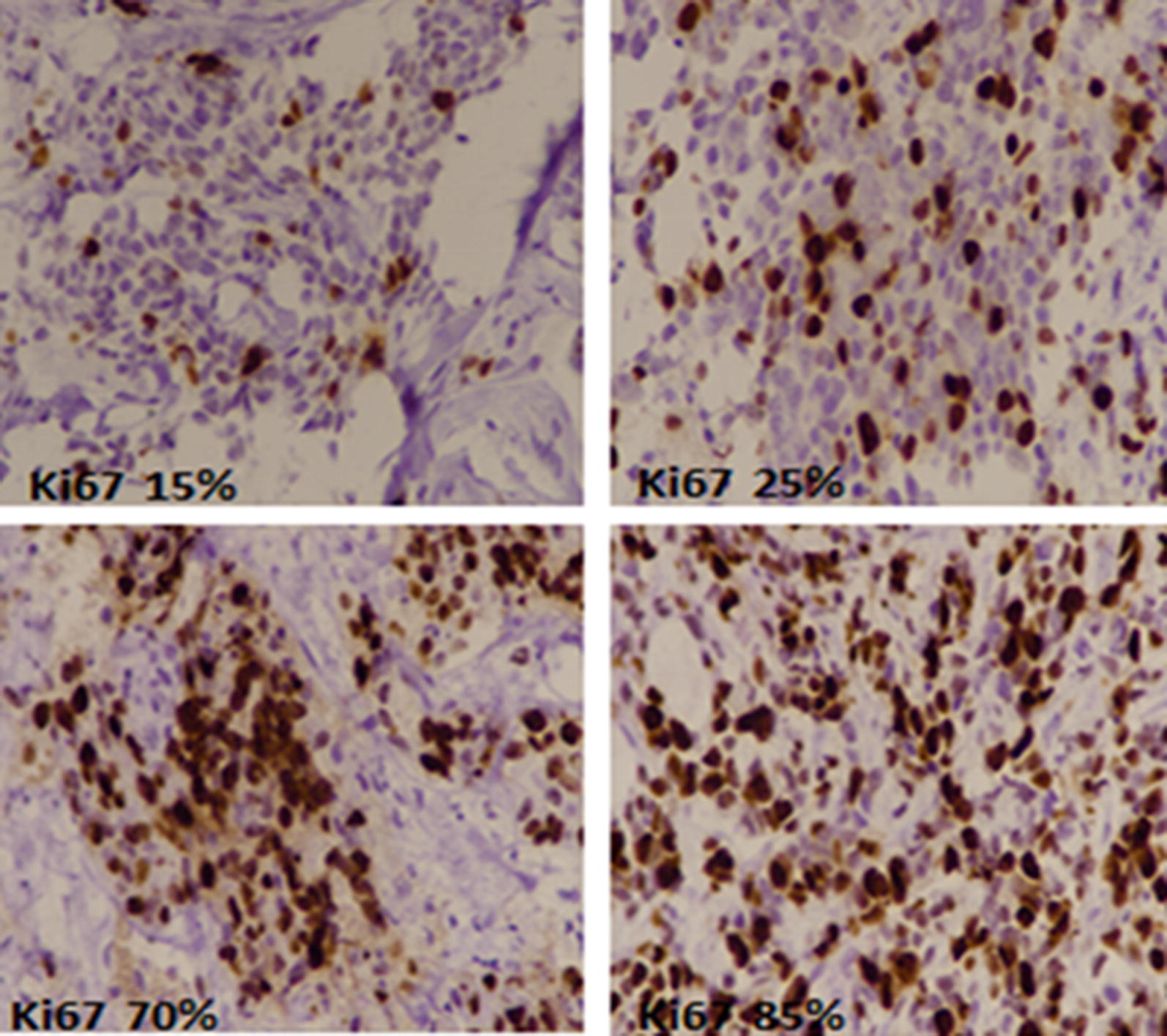
Ki67 expression in breast cancer by immunohistochemistry. Brown nuclear stain highlights ki67 positive tumor cells

Histopathologic characteristics including histologic type, grade, tumor size, nodal status, lymphocytic infiltration of tumor, necrosis and fibrosis were assessed by two histopathologists independently. One representative section was selected for IHC studies including ER, PR, her2neu and ki67. Antibodies for ER, PR and Her2neu IHC were purchased from DAKO and DAKO envision kit was used and stains were performed according to manufacturer’s defined protocol. Positive and negative controls were run along each batch of IHC. Only nuclear expression of ER and PR were recorded semi-quantatively and more than 1% expression was taken as positive expression [6, 7]. For Her2neu IHC, only membranous staining was considered and more than 10% strong membranous positivity was taken as positive (3+) Her2neu IHC as per CAP guidelines [8, 9]. Cases with equivocal (2+) IHC expression of Her2neu subsequently underwent FISH testing for Her2neu gene amplification. FISH testing was done using Path Vysion Her2 DNA Probe kit. Results were interpreted as amplified (positive) or not amplified (negative) according to CAP guidelines [8, 9]. For Ki-67, nuclear expression was recorded quantitatively. At-least 1000 cells were assessed to calculate an average estimate. On the basis of percentage of staining, ki67 index was further categorized into four groups, < 14%, 15–24%, 25–44%, > 45%, as can be seen in Fig. 1.

Surrogate clinicopathologic definitions of intrinsic breast cancer subtypes were used as follows [10]:

1. Luminal A like: ER positive, PR high (> 20%).
2. Luminal B like: ER positive, PR low (< 20%), or ER positive, Her2neu positive (3 + on IHC/amplified on FISH), any PR.
3. Her2neu positive (non-luminal): ER and PR negative, Her2neu positive (3+ on IHC or amplified on FISH (for 2+ IHC results)
4. Triple negative: ER, PR and Her2neu negative.

For data analysis, Statistical package for social sciences (SPSS 21) was used. Mean and standard deviation were evaluated for quantitative variables. Frequency and percentage were evaluated for qualitative variables. Chi square and fisher exact test was applied to determine association as appropriate. ANOVA was applied to compare difference in means among groups. *P* value ≤ 0.05 was considered significant.

### Results

**Fig. 2:**
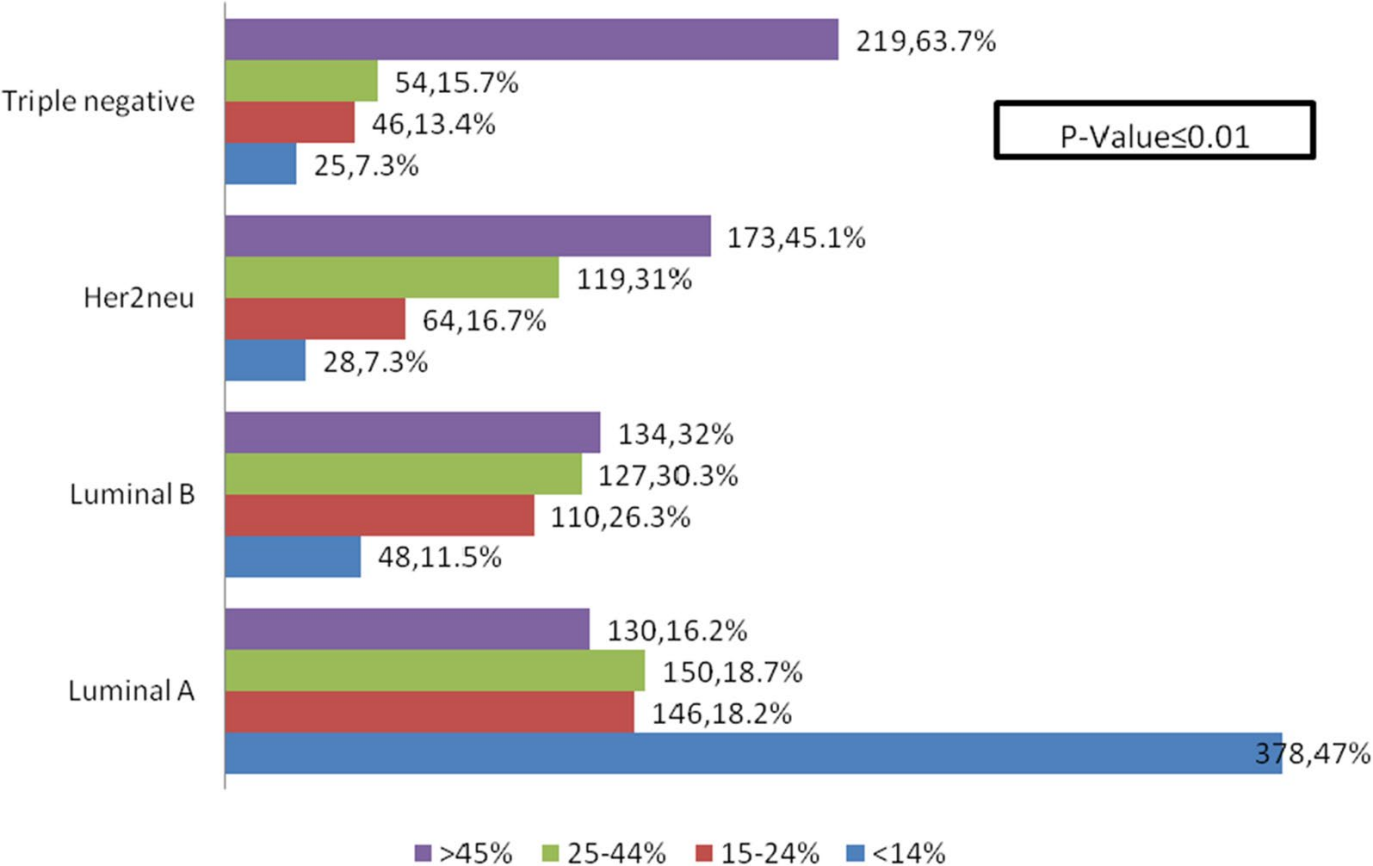
Ki67 index expression in different intrinsic breast cancer subtypes, categorized into 4 sub-groups and shown at the bottom of the figure

Out of total 1951 cases of primary breast cancers included in the study, 1185 cases were of trucut biopsies while 766 cases were excision specimens. Figure 2 shows association of ki67 index with intrinsic breast cancer subtypes. Triple negative breast cancers showed highest ki67 index (mean 50.9 ± 23.7%) followed by Her2neu (mean 42.6 ± 21.6%) and luminal B cancers (mean 34.9 ± 20.05%). On the other hand, luminal A cancers showed lowest ki67 index (mean 23.6 ± 19.7%). Table 1 depicts association of ki67 index categories with histologic subtypes. Metaplastic and medullary breast cancers significantly showed higher ki67 index as compared to ductal carcinoma, NOS.

**Table 1:** Association of ki67 index with Histological subtypes

Histologic subtype	Ki67 index category N (%)				Total	P-value
	< 15%	15–24%	25–44%	> 44%		
Ductal	373 (22)	311 (18.3)	406 (24)	605 (36)	1695	< 0.01
Lobular	46 (50.5)	20 (22)	13 (14.3)	12 (13.2)	91	
Cribriform	4 (57.1)	2 (28.6)	1 (14.3)	0 (0)	7	
Papillary	19 (47.5)	10 (25)	6 (15)	5 (12.5)	40	
Mucinous	23 (63.9)	6 (16.7)	5 (13.9)	2 (5.6)	36	
Micropapillary	2 (13.3)	4 (26.7)	5 (33.3)	4 (26.7)	15	
Tubular	7 (70)	0 (0)	1 (10)	2 (20)	10	
Medullary	0 (0)	0 (0)	1 (11.1)	8 (88.9)	9	
Metaplastic	4 (9.3)	11 (25.6)	12 (28)	16 (37.2)	43	
Mixed Ductal &Lobular	1 (25)	1 (25)	0 (0)	2 (50)	4	
Adenoid cystic carcinoma	0 (0)	1 (100)	0 (0)	0 (0)	1	
Total	479	366	450	656	1951	

Fisher exact test was applied

Additional file 1: Tables S1–S4 shows association of ki67 index with various clinical and pathologic parameters according to different subtypes of breast cancer. ki67 showed significant association with tumor grade in all breast cancer subtypes.

Significant association of ki67 index was also seen with age in triple negative and luminal A subtypes. Higher ki67 index was noted in lower age groups specifically < 30 years age group. No significant association of ki67 index was noted with any of the other histological parameters or nodal stage.

### Discussion

In the present study, we evaluated ki67 index in different intrinsic and histologic breast cancer subtypes and found high ki67 index in her2neu and triple negative intrinsic breast cancer subtype and metaplastic & medullary histologic breast cancer types [11, 12]. All of these categories of breast cancer are uniformly considered as aggressive phenotypes of breast cancer. Moreover, significant association of ki67 index was noted with tumor grade which is considered as one of the prognostic factor in breast cancer [13, 14]. Apart from its association with tumor grade, we didn’t find any significant association of ki67 index with any other prognostic parameter including nodal metastasis. Furthermore, we also found a significantly high ki67 index (> 44%) in women < 30 years of age in triple negative and luminal B subtypes. A high frequency of young age breast cancer has been reported in previous studies conducted in this part of the world [15]. Although, lack of availability of widespread molecular tests makes it difficult to identify the genomic profile of young age breast cancer in our population; nevertheless, importance of these findings can’t be overlooked.

The association of ki67 index with prognostic profile of breast cancer has been extensively studied [16, 17]. Despite inconsistency in defining cutoff values and lack of inter-laboratory validity in ki67 results, it has been shown that ki67 index is an independent prognostic factor in breast cancer. Results of a large meta-analysis involving 64,196 patients concluded that; when using > 25% ki67 (as high ki67 index) cutoff, ki67 index is an independent prognostic factor in terms of overall survival in breast cancer patients [18]. Similarly, a meta-analysis analyzed samples from randomized controlled trials and confirmed the independent prognostic value of ki67 [19]. Another meta-analysis included 46 studies and 12,155 patients; they reported that high ki67 was associated with higher risk of relapse in both node negative and node positive disease and worse survival in breast cancer [20]. We didn’t evaluate the survival and recurrence status of patients in our study which was one of the limitations of our study.

Ki67 index in different molecular subtypes of breast cancer has been investigated in various studies. Soliman et al. reported a high ki67 index (> 15%) in 34% & 60% of her2neu and triple negative breast cancer respectively [21]. On the other hand, we found an even high ki67 in these two subtypes of breast cancer; more than 90% of her2neu and triple negative breast cancers had ki67 > 14% in our study.

St. Gallen international expert consensus on primary therapy for early breast cancer 2013, defined surrogate clinicopathologic definitions of intrinsic breast cancer subtypes taken into account percentage of PR positivity (cutoff > 20%) and ki67 index. There was a disagreement on the exact cutoffs for ki67 index. Although a cutoff value of 20% was proposed, especially for the adjuvant use of chemotherapy; however cutoff value of 14% beast correlated with gene expression definition of luminal A breast cancer [10].

### Conclusion

Ki67 index is a valuable biomarker of breast cancer as higher ki67 correlates with higher tumor grade. However, no independent prognostic significance of ki67 index could be established in our study due to lack of its association with nodal metastasis or any other prognostic factor in breast cancer.

## Limitations

One of the major limitations of our study was that, recurrence status of patients was not evaluated; therefore, we recommend more large-scale studies evaluating prognostic significance of ki67 in terms of tumor recurrence and disease-free survival.

## Supplementary information

**Additional file 1.** Additional tables.

## Data Availability

Please contact author for data requests.

## Abbreviations

ER: estrogen receptor
PR: progesterone receptor
ASCO: American Society of Clinical Oncology
