# Supplementary material for: Ki67 index in intrinsic breast cancer subtypes and its association with prognostic parameters
Source: BMC Res Notes. 2019 Sep 23;12:605. doi: 10.1186/s13104-019-4653-x (PMC6755684; doi:10.1186/s13104-019-4653-x)
Supplement: Supplementary file 1 — Additional file 1. Additional tables. [file 13104_2019_4653_MOESM1_ESM.doc]

**Table S1: Association of ki67 index with clinico-pathologic parameters of triple negative breast cancer subtype**

| **Clinicopathological Parameters** | **Ki67 index category N (%)** | | | | **P-value** |
| --- | --- | --- | --- | --- | --- |
|  | **<15%** | **15-24%** | **25-44%** | **>44%** |  |
|  |  |  |  |  |  |
| **Age(years)±** | 49.16±14.43 | 51.83±12.09 | 52.02±12.13 | 46.64±11.95 | <0.01 |
| **Age Group** |  |  |  |  |  |
| <30 | 3(10.7) | 0(0) | 1(3.6) | 24(85.7) | <0.05 |
| 30-50 | 11(6.2) | 23(12.9) | 30(16.9) | 114(64) |  |
| 51-70 | 8(6.5) | 20(16.3) | 20(16.3) | 75(61) |  |
| >70 | 3(20) | 3(20) | 3(20) | 6(40) |  |
| **T Stage** |  |  |  |  |  |
| T1 | 2(11.1) | 2(11.1) | 7(38.9) | 18 | 0.644 |
| T2 | 10(9.8) | 18(17.6) | 19(18.6) | 102 |  |
| T3 | 2(9.1) | 3(13.6) | 6(27.3) | 22 |  |
| **N Stage** |  |  |  |  |  |
| N0 | 10(11.6) | 11(12.8) | 21(24.4) | 44(51.2) | 0.369 |
| N1 | 1(5.6) | 3(16.7) | 1(5.6) | 13(72.2) |  |
| N2 | 0(0) | 1(12.5) | 2(25) | 5(62.5) |  |
| N3 | 3(10) | 8(26.7) | 8(26.7) | 11(36.7) |  |
| **Tumor Grade** |  |  |  |  |  |
| Grade I | 4(28.6) | 1(7.1) | 0(0) | 9(64.3) | <0.01 |
| Grade II | 8(8.2) | 22(22.4) | 15(15.3) | 53(54.1) |  |
| Grade III | 13(5.6) | 23(9.9) | 39(16.8) | 157(67.7) |  |
| **Lymphovascular Invasion** |  |  |  |  |  |
| Present | 3(11.1) | 5(18.5) | 7(25.9) | 12(44.4) | 0.837 |
| Absent | 11(9.6) | 18(15.7) | 25(21.7) | 61(53) |  |
|  |  |  |  |  |  |
| ± Mean±SD, ANOVA were applied. |  |  |  |  |  |
| Fisher exact test was applied. |  |  |  |  |  |

**Table S2: Association of ki67 index with clinico-pathologic parameters of Her2neu breast cancer subtype**

| **Clinicopathological Parameters** | **Ki67 index category N (%)** | | | | **P-value** |
| --- | --- | --- | --- | --- | --- |
|  | **<15%** | **15-24%** | **25-44%** | **>44%** |  |
|  |  |  |  |  |  |
| **Age(years)±** | 49.79±13.06 | 51.70±12.17 | 47.43±10.73 | 49.76±11.72 | 0.107 |
| **Age Group** |  |  |  |  |  |
| <30 | 3(20) | 2(13.3) | 6(40) | 4(26.7) | 0.116 |
| 30-50 | 9(4.4) | 31(15.1) | 67(32.7) | 98(47.8) |  |
| 51-70 | 14(9.5) | 27(18.2) | 44(29.7) | 63(42.6) |  |
| >70 | 2(12.5) | 4(25) | 2(12.5) | 8(50) |  |
| **T Stage** |  |  |  |  |  |
| T1 | 2(14.3) | 2(14.3) | 3(21.4) | 7(50) | 0.452 |
| T2 | 4(4.9) | 14(17.3) | 30(37) | 33(40.7) |  |
| T3 | 3(11.5) | 6(23.1) | 10(38.5) | 7(26.9) |  |
| **N Stage** |  |  |  |  |  |
| N0 | 5(8.9) | 11(19.6) | 18(32.1) | 22(39.3) | 0.407 |
| N1 | 0(0) | 0(0) | 8(47.1) | 9(52.9) |  |
| N2 | 1(5.6) | 5(27.8) | 5(27.8) | 7(38.9) |  |
| N3 | 3(10) | 6(20) | 12(40) | 9(30) |  |
| **Tumor Grade** |  |  |  |  |  |
| Grade I | 2(25) | 3(37.5) | 1(12.5) | 2(25) | <0.01 |
| Grade II | 19(9.3) | 42(20.6) | 70(34.3) | 73(35.8) |  |
| Grade III | 7(4.1) | 19(11) | 48(27.9) | 98(57) |  |
| **Lymphovascular Invasion** |  |  |  |  |  |
| Present | 4(11.4) | 8(22.9) | 14(40) | 9(25.7) | 0.218 |
| Absent | 5(5.8) | 14(16.3) | 29(33.7) | 38(44.2) |  |
|  |  |  |  |  |  |
| ± Mean±SD, ANOVA were applied. |  |  |  |  |  |
| Fisher exact test was applied. |  |  |  |  |  |

**Table S3: Association of ki67 index with clinico-pathologic parameters of Luminal A breast cancer subtype**

| **Clinicopathological Parameters** | **Ki67 index category N (%)** | | | | **P-value** |
| --- | --- | --- | --- | --- | --- |
|  | **<15%** | **15-24%** | **25-44%** | **>44%** |  |
|  |  |  |  |  |  |
| **Age(years)±** | 54.61±12.77 | 54.95±12.65 | 53.57±12.51 | 49.21±12.14 | <0.01 |
| **Age Group↨** |  |  |  |  |  |
| <30 | 14(41.2) | 6(17.6) | 5(14.7) | 9(26.5) | <0.05 |
| 30-50 | 139(43.7) | 47(14.8) | 68(21.4) | 64(20.1) |  |
| 51-70 | 192(49.1) | 78(19.9) | 66(16.9) | 55(14.1) |  |
| >70 | 33(54.1) | 14(24.6) | 11(18) | 2(3.3) |  |
| **T Stage↨** |  |  |  |  |  |
| T1 | 35(58.3) | 15(25) | 7(11.7) | 3(5) | 0.723 |
| T2 | 138(53.1) | 61(23.5) | 33(12.7) | 28(10.8) |  |
| T3 | 30(55.6) | 9(16.7) | 8(14.8) | 7(13) |  |
| **N Stage** |  |  |  |  |  |
| N0 | 114(56.4) | 48(23.8) | 25(12.4) | 15(7.4) | 0.097 |
| N1 | 50(52.1) | 18(18.8) | 14(14.6) | 14(14.6) |  |
| N2 | 16(40) | 14(35) | 3(7.5) | 7(17.5) |  |
| N3 | 23(63.9) | 5(13.9) | 6(16.7) | 2(5.6) |  |
| **Tumor Grade↨** |  |  |  |  |  |
| Grade I | 113(69.3) | 24(14.7) | 18(11) | 8(4.9) | <0.01 |
| Grade II | 233(46.5) | 97(19.4) | 96(19.2) | 75(15) |  |
| Grade III | 32(22.9) | 25(17.9) | 36(25.7) | 47(33.6) |  |
| **Lymphovascular Invasion↨** |  |  |  |  |  |
| Present | 31(43.1) | 17(23.6) | 10(13.9) | 14(19.4) | <0.05 |
| Absent | 172(57) | 68(22.5) | 38(12.6) | 24(7.9) |  |
|  |  |  |  |  |  |
| ± Mean±SD, ANOVA were applied. |  |  |  |  |  |
| Fisher exact test was applied. |  |  |  |  |  |
| ↨Chi-Square Test was applied. |  |  |  |  |  |

**Table S4: Association of ki67 index with clinico-pathologic parameters of Luminal B breast cancer subtype**

| **Clinicopathological Parameters** | **Ki67 index category N (%)** | | | | **P-value** |
| --- | --- | --- | --- | --- | --- |
|  | **<15%** | **15-24%** | **25-44%** | **>44%** |  |
|  |  |  |  |  |  |
| **Age(years)±** | 53.29±13.30 | 50.09±12.89 | 49.13±11.62 | 48.24±12.10 | 0.100 |
| **Age Group** |  |  |  |  |  |
| <30 | 3(13.6) | 6(27.3) | 6(27.3) | 7(31.8) | 0.225 |
| 30-50 | 16(7.4) | 56(25.9) | 66(30.6) | 78(36.1) |  |
| 51-70 | 24(15.2) | 42(26.6) | 51(32.3) | 41(25.9) |  |
| >70 | 5(21.7) | 6(26.1) | 4(17.4) | 8(34.8) |  |
| **T Stage** |  |  |  |  |  |
| T1 | 4(25) | 7(43.8) | 2(12.5) | 3(18.8) | 0.600 |
| T2 | 12(12.8) | 30(31.9) | 28(29.8) | 24(25.5) |  |
| T3 | 4(21.1) | 5(26.3) | 5(26.3) | 5(26.3) |  |
| **N Stage** |  |  |  |  |  |
| N0 | 9(17) | 21(39.6) | 16(30.2) | 7(13.2) | 0.171 |
| N1 | 6(21.4) | 8(28.6) | 4(14.3) | 10(35.7) |  |
| N2 | 3(11.5) | 5(19.2) | 8(30.8) | 10(38.5) |  |
| N3 | 2(9.1) | 8(36.4) | 7(31.8) | 5(22.7) |  |
| **Tumor Grade** |  |  |  |  |  |
| Grade I | 13(30.2) | 11(25.6) | 9(20.9) | 10(23.3) | <0.01 |
| Grade II | 30(11.5) | 82(31.4) | 75(28.7) | 74(28.4) |  |
| Grade III | 5(4.3) | 17(14.8) | 43(37.4) | 50(43.5) |  |
| **Lymphovascular Invasion↨** |  |  |  |  |  |
| Present | 7(14.9) | 11(23.4) | 14(29.8) | 15(31.9) | 0.306 |
| Absent | 13(15.9) | 31(37.8) | 21(25.6) | 17(20.7) |  |
|  |  |  |  |  |  |
| ± Mean±SD, ANOVA were applied. |  |  |  |  |  |
| Fisher exact test was applied. |  |  |  |  |  |
| ↨Chi-Square Test was applied. |  |  |  |  |  |
